# Field Evaluation of Rice Husk Biochar and Pine Tree Woodchips for Removal of Tire Wear Particles from Urban Stormwater Runoff in Oxford, Mississippi (USA)

**DOI:** 10.3390/su17094080

**Published:** 2025-04-30

**Authors:** Boluwatife S. Olubusoye, James V. Cizdziel, Kendall Wontor, Ruojia Li, Rachel Hambuchen, Voke Tonia Aminone, Matthew T. Moore, Erin R. Bennett

**Affiliations:** 1 Department of Chemistry and Biochemistry, University of Mississippi, University, MS 38677, USA; 2 Department of Biomolecular Sciences, University of Mississippi, University, MS 38677, USA; 3 Water Quality and Ecology Research Unit, Agricultural Research Service, U.S. Department of Agriculture, Oxford, MS 38655, USA; 4 School of the Environment, Trent University, Peterborough, ON K9L 0G2, Canada

**Keywords:** tire and road wear particles, microplastics, water pollution, sustainable mitigation, aquatic ecosystem, biofilters

## Abstract

Tire wear particles (TWPs), a form of microplastics (MPs) pollution, are transported into waterbodies through stormwater runoff, leading to environmental pollution and impacts on associated biota. Here, we investigated the effectiveness of stormwater filter socks filled with rice husk biochar or pine tree woodchips in reducing TWP pollution in urban runoff in Oxford, Mississippi. Triplicate runoff samples were collected upstream and downstream of the biofilters at two sites during two storm events at peak flow within minutes of the start of the storm and after 30 min. Samples were analyzed for TWPs using a combination of stereomicroscopy, micro-attenuated total reflectance Fourier transform infrared spectroscopy (μ-ATR-FTIR), and scanning electron microscopy (SEM) with energy dispersive X-ray spectroscopy (EDX). Concentrations (TWPs/L) upstream of the biofilter were variable but highest at the start of the runoff, dropping from an average of 2811 ± 1700 to 476 ± 63 after 30 min at site 1 and from 2702 ± 353 to 2356 ± 884 at site 2. Biochar was more effective than woodchips (*p* < 0.05) at removing TWPs, reducing concentrations by an average of 97.6% (first use) and 85.3% (second use) compared to 66.2% and 54.2% for woodchips, respectively. Biochar was particularly effective at removing smaller TWPs (<100 μm). Both materials became less effective with use, suggesting fewer available trapping sites and the need for removal and replacement of the material with time. Overall, this study suggests that biochar and woodchips, alone or in combination, deserve further scrutiny as a potential cost-effective and sustainable method to mitigate the transfer of TWPs to aquatic ecosystems and associated biota.

## 1. Introduction

Microplastic (MP) pollution is of increasing public interest and concern [1–3]. As a result, research has aimed to better quantify their sources, occurrence, and impacts in the environment and in organisms, including humans [4–7]. Their presence in aquatic environments is a global concern due to their abundance and persistence and because they can also adsorb and transport pollutants, such as heavy metals and persistent organic pollutants, increasing their potential toxicity [8,9].

Tire wear particles (TWPs) are estimated to be one of the largest sources of MPs entering the aquatic environment [10–12]. TWPs are generally classified as a subset of MPs because of their size ranges and are primarily composed of natural and synthetic rubber along with carbon black, silica, and other chemical additives [13–15]. TWPs are generated through the abrasion and friction of tires against road surfaces [16,17] and are typically dark or black. They often exhibit elongated or cylindrical shapes with a rough surface texture, may have imbedded road particles, and can range in size from ~1 μm to over 100 μm [13,18,19].

TWPs are a major source of airborne particulate pollution in urban environments, contributing significantly to MPs, both in particle number and mass concentrations [20]. Research suggests that a reduction in TWP emissions is unlikely soon [18,21]. TWPs are also a significant source of pollution to aquatic ecosystems, as they are readily mobilized by precipitation, predominantly through urban runoff, transporting them to downstream surface waters [8,17,21,22]. Recent studies have characterized and quantified TWPs in various environmental compartments, including air [23,24], soil [25], stormwater runoff [19], tributaries [26,27], and snowbanks [28].

Road runoff laden with TWPs, oil, and other pollutants poses a threat to water quality and is widely regarded as a significant non-point source of pollutants to aquatic ecosystems [19,29,30]. The effects of TWPs on aquatic organisms may stem from the TWPs themselves or from compounds released from them [17,31–34]. Of particular concern is the antioxidant N-(1,3-dimethylbutyl)-N’-phenyl-p-phenylenediamine (6PPD), which is widely used in tire rubber to prevent cracking, resist thermal oxidative degradation, and extend tire lifespan [35]. 6PPD reacts with ozone in the environment to form 6PPD-quinone (6PPD-Q), a toxic compound that has been identified in natural waterbodies [27,32,36], roadway runoff [37], and wastewater [38]. 6PPD-Q is toxic to coho salmon (*Oncorhynchus kisutch*), capable of causing acute mortality [32], and it has been linked to reduced survival and reproduction rates in *Daphnia pulex* [30] as well as acute toxicity in *Daphnia magna* [39]. In addition to these toxic effects across a range of species, 6PPD-Q bioaccumulates in organisms, which raises concerns about its impacts on those at higher levels in the food chain, including humans [35].

Therefore, it is essential to mitigate the impact of TWPs and their leachates on aquatic life. Although studies have examined methods to reduce MPs in runoff using, for example, bioretention cells [40], sand filters [41], or bark and biochar filters [42,43], most involve column studies under controlled laboratory conditions. Few investigations have explored mitigating TWP pollution in stormwater runoff under natural (field) conditions. Bioretention cells and stormwater management ponds can be effective in reducing downstream concentrations of MPs and other particulate matter in stormwater, particularly for larger particles (>100 μm) [40,44,45]. However, the effectiveness of these systems decreases with particle size [44], which poses a challenge for removing TWPs, most of which are <100 μm [13,46]. Thus, there continues to be a need for cost-effective strategies to minimize the transport of TWPs from stormwater runoff into aquatic ecosystems.

Biochar has been recognized as a promising material for contaminant removal in aqueous systems [47–49]. It offers several advantages, including a large specific surface area, abundant functional groups, high stability, strong adsorption capacity, low cost, and well-developed pore structure [50]. Specifically, biochar produced at elevated temperatures typically exhibits a high surface area, increased carbon content, and enhanced micropore volume [51]. These physicochemical properties make biochar an effective adsorbent for contaminants [52–54]. For instance, pinewood biochar pyrolyzed at 900 °C has demonstrated a 92% retention rate of microplastics (MPs) in agricultural runoff [43]. Additionally, activated biochar has been shown to remove MPs in aqueous systems [55], and biochar filters have proven effective in immobilizing 10 μm MP spheres [53]. However, bark has also been investigated for MP removal [42]. Due to its porosity and rich composition of natural organic compounds (including cellulose, polysaccharides, lignin, and tannins), bark has been utilized in filtration studies for contaminant removal [56,57]. Similarly, wood residues coated with natural phenols effectively removed a diverse mixture of nanoplastics (NPs) and small-sized MPs in flow-through vertical filters [58].

In this pilot study, we evaluated the effectiveness of rice husk biochar and pinewood woodchips as low-cost media to filter out TWPs from urban stormwater runoff under field conditions for two separate sites and for two storm events. Stormwater filter socks filled with the biochar or woodchips were positioned such that runoff from roadways and parking lots passed through them, and TWP concentrations were determined both before and after the biofilters using a combination of stereomicroscopy, μ-ATR-FTIR, and SEM-EDX for characterization and quantitation. Given the similar characteristics with bark and wood residues, we hypothesize that woodchips (rich in cellulose and lignin) may offer similar filtration capabilities that can be explored for potential and sustainable TWP removal. To the author’s knowledge, this is the first study to evaluate the effectiveness of biochar and woodchips in filter socks to remove TWPs from urban runoff using these field and analytical approaches, and it encourages broader adoption of sustainable stormwater filtration systems that can lead to significant reductions in roadway pollution transferring to waterbodies.

## 2. Materials and Methods

### 2.1. Study Area

**Figure 1.**
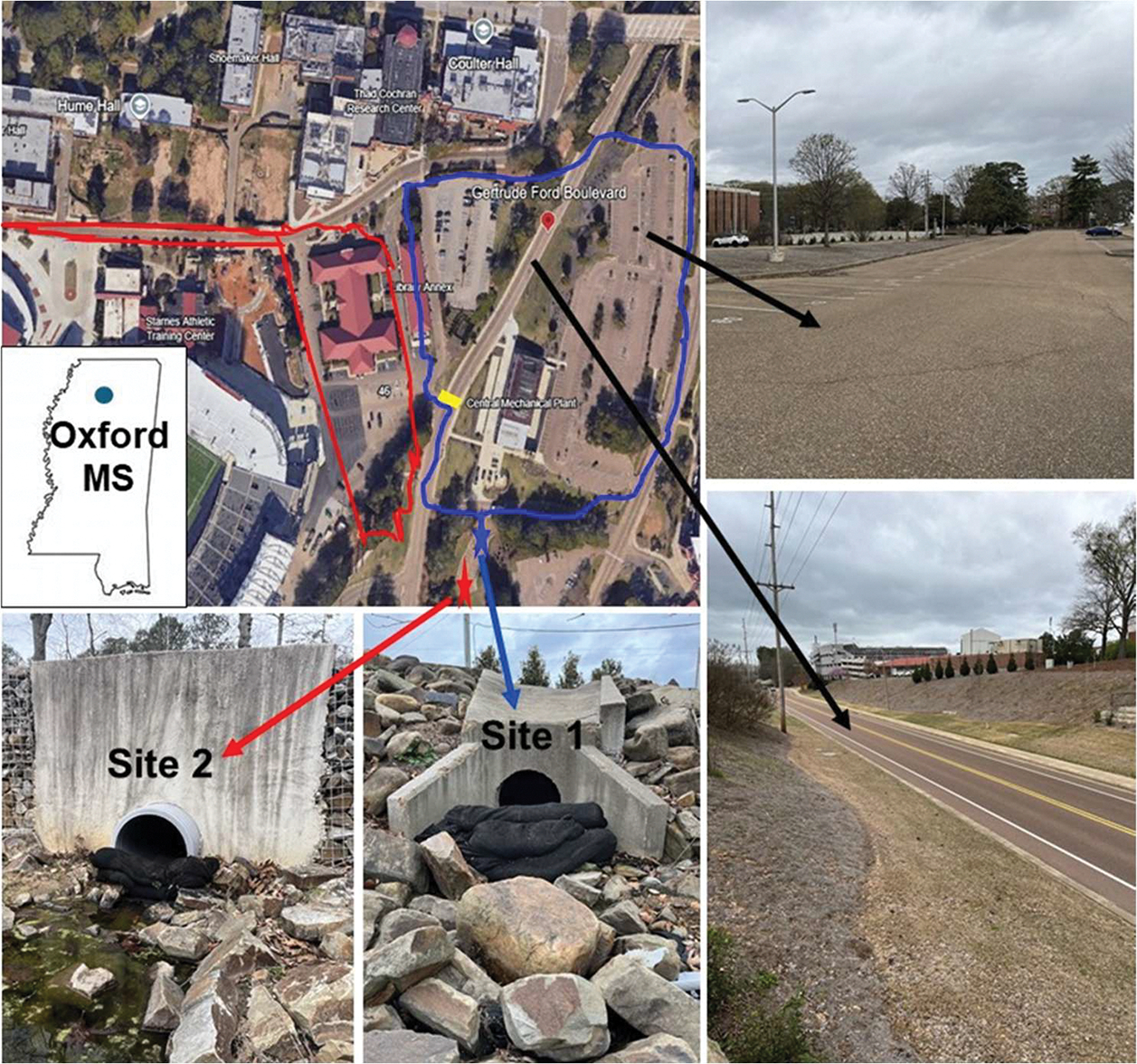
Study site showing (1) the location of Oxford in Mississippi (inset), (2) an aerial view showing the sampling sites (blue star = site 1, red star = site 2) and the approximate drainage area for each (top left), (3) photos of the sampling sites with the filter socks in place (bottom), and (4) photos of a drainage area 1 including a portion of the parking lot and Gertrude Ford Boulevard (right). Note, a pedestrian crosswalk is highlighted in yellow in the center image, and the drainage for area 2 (red) is piped under the road to exit at sampling site 2. Photos taken by the authors (J.V.C.).

Two locations were selected for sampling, both of which are adjacent to the University of Mississippi (UM) campus in Oxford, MS, USA, with a combined city and student population of ~55,000 (Figure 1). The sites include runoff from campus parking lots and Gertrude Ford Boulevard (GFB), a campus thoroughfare and city access road with moderate traffic volume (~12,000 vehicles daily) and moderate traffic speed (average vehicular speed of ~65 km/h) [23]. In a previous study, we quantified airborne putative TWPs in the same study area and reported deposition rates of 184 ± 93 particles/cm^2^/day (mean ± SD). Our findings revealed that TWP abundance, both in terms of deposition and mass concentration, increased with vehicle braking [23]. The GFB road includes pedestrian walkways between parking lots that can cause motorists to apply brakes, generating TWPs [23,59], making it an ideal area for the present study. At site 1, runoff is funneled from a total paved area of ~35,147 m^2^, with 85% of the surface areas from parking lots and 15% from GFB (roadway), whereas at site 2, runoff is funneled from ~17,745 m^2^, with 88% from parking lots and 12% from a low traffic road on campus (Figure 1).

### 2.2. Biofilter Preparation and Sample Collection

Rice husk biochar was obtained from the Mississippi Agricultural and Forestry Experiment Station at Mississippi State University. The physical and chemical characteristics of the biochar are given in Table 1. Pine tree woodchips were obtained from the USDA National Sedimentation Laboratory in Oxford, MS. The woodchips averaged 3–5 cm in size. Both the rice husk biochar and woodchips were packed into separate filter socks measuring ~25 cm in width and ~3 m length. The filter socks were then positioned at stormwater drainage outlets along GFB. Triplicate samples were collected rapidly one after another (all within <1 min) in 1 L mason jars, first upstream and then downstream of the filter socks at each sampling site, during storm events on 26 June 2024 and 25 July 2024 (Table 2). Samples were taken within minutes of the start of the storm, during peak flow, and again after 30 min, with diminished flow but with rain still falling. The first storm event on 26 June 2024 lasted for about 45 min, while the second event on 25 July 2024 lasted for about 1 h. Since the first storm event lasted ~45 min, sampling during the second storm event was discontinued after 30 min of runoff treatment to allow for a consistent comparison between both events. Upstream samples were collected within the outlet pipe where water was flowing unrestricted without any backflow, and downstream samples were collected where the flow funneled for easy sampling. Flow rates were estimated by timing the filling of each sampling jar. For the second storm event, the same filter socks were used but their positions were swapped (i.e., the biochar was moved from site 1 to site 2 and the woodchips from site 2 to site 1). This was performed, in part, to compare the results for the two biofilters for the same drainage area, as the characteristics for each were different (Section 2.1). Prior to the second storm event, the filter socks were sun-dried in the field for approximately one month before being swapped and testing their effectiveness in reuse.

### 2.3. Isolation and Preconcentration of TWPs

Samples were transported to the laboratory for analysis within an hour of collection, stored at 4 °C, and processed within a week. TWPs were isolated using the “one pot” method, whereby both sample digestion and density separation occur in the same jar the sample was collected in, thus minimizing contamination and sample loss [61]. Briefly, triplicate samples were passed through a Monel screen (200 × 600 mesh, ~30 μm openings) to remove the water. Particles >30 μm left on the screen were then carefully rinsed back into the glass jar using 0.22 μm filtered Milli-Q water (Millipore, Burlington, NJ, USA). The mixture was then subjected to digestion using Fenton’s reagent (30 mL of 30% hydrogen peroxide and 15 mL of 0.05 M iron (II) sulfate in 0.6% sulfuric acid) at room temperature to remove natural organic matter without damaging the TWPs. This was followed by density separation using ~100 mL of ZnCl_2_ (1.6 g cm^−3^) to further isolate the TWPs by removing heavier particles such as sand. Whereas the density of directly abraded tire tread is ~1.13–1.16 g cm^−3^ [62], most TWPs along roads have densities between 1.3 and 1.7 g cm^−3^ due, in part, to imbedded road, sand, or other particles [18,46,63]. Thus, a ZnCl_2_ solution with a density of 1.6 g cm^−3^ will float a substantial majority, but not all, TWPs. Here, floating particles were collected from the surface of the ZnCl_2_ solution with a glass pipette and transferred into a clean glass vial. Afterward, the solution was mixed and allowed to sit overnight before collecting particles on the surface again, and this process was repeated for a total of three collections. Collected particles were then vacuum filtered onto 25 mm × 25 mm polycarbonate filters with 10 μm pores (Isopore Membrane, Sigma Aldrich, Saint Louis, MO, USA). The filters were carefully transferred into a petri slide and allowed to dry before covering them until observation and analysis.

### 2.4. Analysis of TWPs by Stereomicroscopy, μ-ATR-FTIR, and SEM-EDX

**Figure 2.**
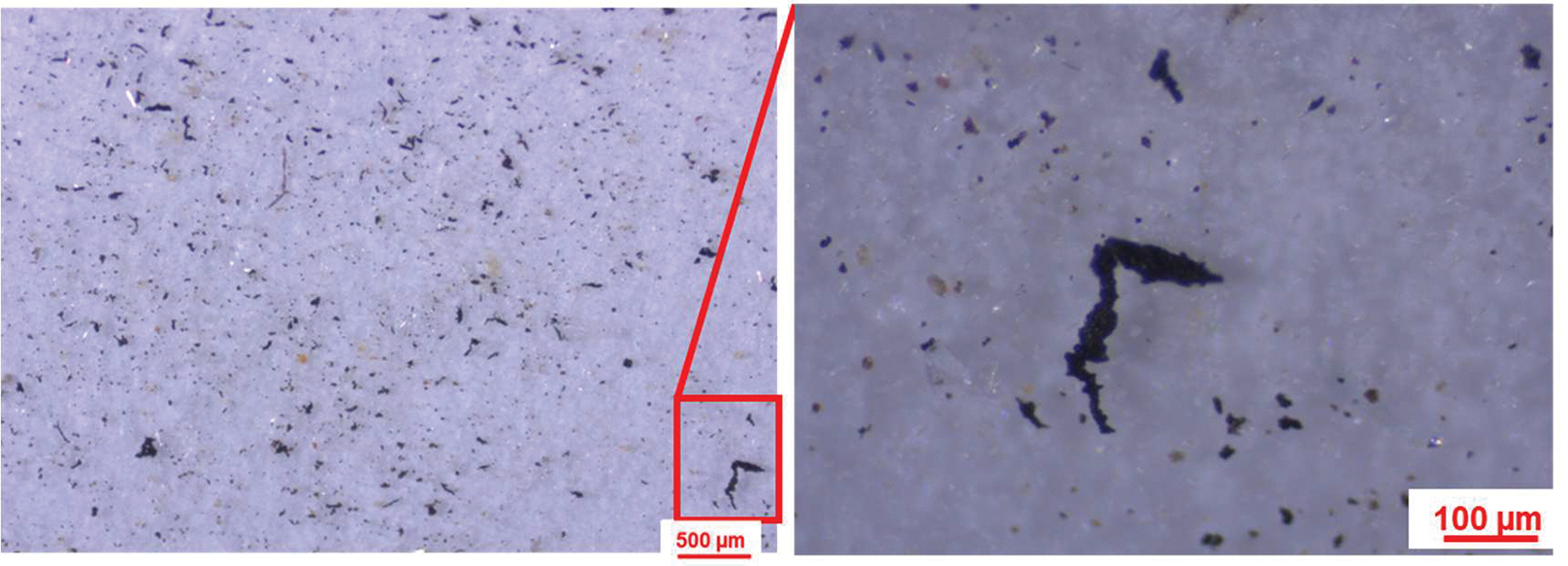
Stereomicroscopic images of TWPs on a polycarbonate filter at 30× magnification (**left**) and 95× magnification (**right**). Zoomed image (**right**) shows a large TWP with distinct features (e.g., black, elongated, jagged-edge, rough surface). Lighter or colored particles and fibers can also be seen.

TWPs can be distinguished by their dark/black color and cylindrical/elongated fragments with rough surface texture or jagged edges [26,47]. Here, TWPs on the filters were visually inspected using a Stemi 508 stereomicroscope equipped with an Axiocam 105 color digital camera (Carl Zeiss, Jena, Germany). Stereomicroscopic images were taken at 30–95× magnification with LED intensity set between 60 and 66 and a resolution of 1.5 μm (See Figure 2). The images were processed by ImageJ (https://imagej.net/ij/) (NIH-LOCI, University of Wisconsin, Madison, WI, USA) for automated particle counting and sizing as described elsewhere [23]. A light intensity threshold of 200 was used to remove non-dark objects, as bright or colored particles are mostly geogenic, biogenic, or non-TWPs. Because the samples were prepared using a 30 μm screen, only particles >30 μm in size were counted; TWPs smaller than this cutoff are generally difficult to distinguish visually [24].

Although TWPs are dark and do not pass or reflect infrared light (making them unable to be analyzed via FTIR in transmission or reflection mode), they have been shown to produce spectra by μ-ATR-FTIR [23,24]. Here, a subset of putative TWPs were analyzed by μ-ATR-FTIR as described in Gao et al. [23]. Briefly, putative TWPs (>30 μm) isolated on the polycarbonate filters (Section 2.3) were carefully transferred using tweezers onto a glass slide under a light microscope. Thereafter, the glass slide was positioned on the LUMOS II FTIR microscope sample stage for single spot analysis using a spectral resolution of 4 cm^−1^ and 16 scans. The LUMOS II system features a retractable ATR crystal, controlled by high-precision piezoelectric motors integrated into the lens, enabling accurate positioning of the crystal on selected particles of interest [23]. Here, spectra were collected from ten putative TWPs using a LUMOS II FTIR microscope with a liquid nitrogen cooled Mercury Cadmium Telluride (MCT) detector and a germanium μ-ATR crystal (Bruker Corp., Billerica, MA, USA). The objective was not to examine every particle but rather to confirm that particles suspected of being TWPs had spectra characteristics of TWPs. The spectra from the particles were matched with the instrument’s spectral libraries (OPUS v8.8.4 software), and all ten putative TWPs returned with isobutylene isoprene rubber as the top match. Our approach demonstrated that the μ-ATR-FTIR can generate high-quality spectra that can aid the identification of TWPs. (See Figure 2).

Putative TWPs, biochar, and woodchips were also examined by SEM and EDX analyses for particle morphology, surface properties, and elemental compositions. As noted, TWPs tend to have features such as jagged edges, rough surfaces, and cylindrical or elongated shapes. They also have an elemental composition, with high levels of carbon and oxygen along with silicon, sulfur, zinc, iron, aluminum, and calcium. Combining these morphological and textural parameters with elemental fingerprints provides a way to recognize and differentiate TWPs [64]. Here, particles from the filter were transferred onto SEM stubs covered with carbon tape by touching the stub to the filter. Ten particles resembling TWPs were then imaged and analyzed by field emission SEM-EDX (JSM-7200FLV, JEOL Ltd., Akishima City, Japan). Samples were sputter-coated prior to analysis with a 60:40 gold to palladium ratio. Analysis was conducted under a vacuum pressure of 10^−4^ Pa, with the electron beam accelerated at 15 kV. Particles of biochar and woodchips were also analyzed in the same manner.

### 2.5. Statistical Analysis

Dataset normality was evaluated using the Shapiro–Wilk test. To determine variations in TWP concentrations before and after the biofilters as well as between the two sampling locations, a non-parametric Kruskal–Wallis one-way ANOVA was conducted. If a statistically significant difference was identified, a Tukey post hoc test was subsequently employed to perform pairwise comparisons between the datasets. All statistical analyses were carried out using Sigma Plot 14.0 (Systat Software, Inc., San Jose, CA, USA), with the significance threshold set at *p* = 0.05. Data visualization and additional analyses, including plot generation, were performed using JMP software v18.2 (SAS Institute Inc., Cary, NC, USA).

### 2.6. Quality Assurance and Control Measures

Precautions were taken to minimize contamination, including wearing cotton laboratory coats dyed bright orange and nitrile gloves during sample preparation and analyses. Samples were prepared in a laminar flow hood inside a HEPA-filtered clean room (AirClean 6000 Workstation, AirClean Systems, Creedmoor, NC, USA). Samples were covered with aluminum foil when not in use or during processing. All glassware and materials were cleaned and rinsed with filtered (0.22 μm) Milli-Q water [43,65]. Blank samples were processed and analyzed alongside the experimental samples, with no TWP contamination detected in the blanks.

## 3. Results and Discussion

### 3.1. Abundance of TWPs in Stormwater Runoff

A total of 37,992 putative TWPs were counted during the study. Table 3 summarizes the data by biofilter type (biochar and woodchip), rain event (26 June 2024 and 25 July 2024), and time (start of runoff and 30 min later). For the raw pre-filtered runoff, the concentrations of TWPs were highly variable from sample to sample but clearly decreased with time for both storm events. The concentrations (TWPs/L; n = 3 samples) were highest at the start of runoff, dropping from 2811 ± 1700 to 476 ± 63 after 30 min at site 1 and from 1765 ± 1037 to 99 ± 36 at site 2 for the first storm event. During the second storm event, the concentrations went from 2255 ± 2064 to 200 ± 67 at site 1 and from 2702 ± 353 to 2356 ± 884 at site 2. It is not unusual for the concentrations of MPs and TWPs in stormwater runoff to peak during the initial phase of runoff and fluctuate with rainfall intensity [66–68]. Runoff from event 1 occurred after 22 days without rainfall, whereas runoff for event 2 was collected following 28 days without rainfall. Combining sites, the TWP concentrations (TWPs/L; n = 6) averaged 2288 ± 1932 at the start of event 1, while the average was slightly higher (2478 ± 1347 TWPs/L) for event 2. This difference may stem from a first flush effect, commonly reported for many pollutants, whereby pollutants accumulate on catchment surfaces with dry days before being washed from the surface with rainfall [69–71]. Concentration differences may also be due to rain intensity and duration, which play a role in pollutant mobility [68,72]. Here, the runoff flow rate (discharge) was higher during the second (July) event (Table 2). Due to the aforementioned factors, it is not surprising that relatively large concentration ranges of TWPs have been observed in roadside soils, sediments, and drainage ditches because of such runoff [25,59,73]. For example, TWP concentrations in roadside soils were 2000–26,400 mg/kg [25], and TWP concentrations in roadside snow were 76.0–14,500 mg/L of meltwater [28].

Varying site characteristics complicate comparisons between sites and studies. Pollutant concentrations in stormwater runoff can vary significantly, both across locations and between storm events, as physical attributes of a drainage area, such as the extent of impervious surfaces and the gradient of the land, play a crucial role in discharge and pollutant transport [68,74]. Here, for both storm events, the average TWP concentrations were generally higher at sampling site 1 (2533 ± 1719 TWPs/L) compared to site 2 (1781 ± 1289 TWPs/L). This difference likely stems from both the larger drainage surface area and the busier road for site 1 compared to site 2. Sampling site 1 covers an estimated paved surface area of ~35,147 m^2^, whereas sampling site 2 spans ~17,745 m^2^. Site 1 also includes GFB, which has much greater traffic volume than the on-campus road within the catchment for site 2.

**Figure 3.**
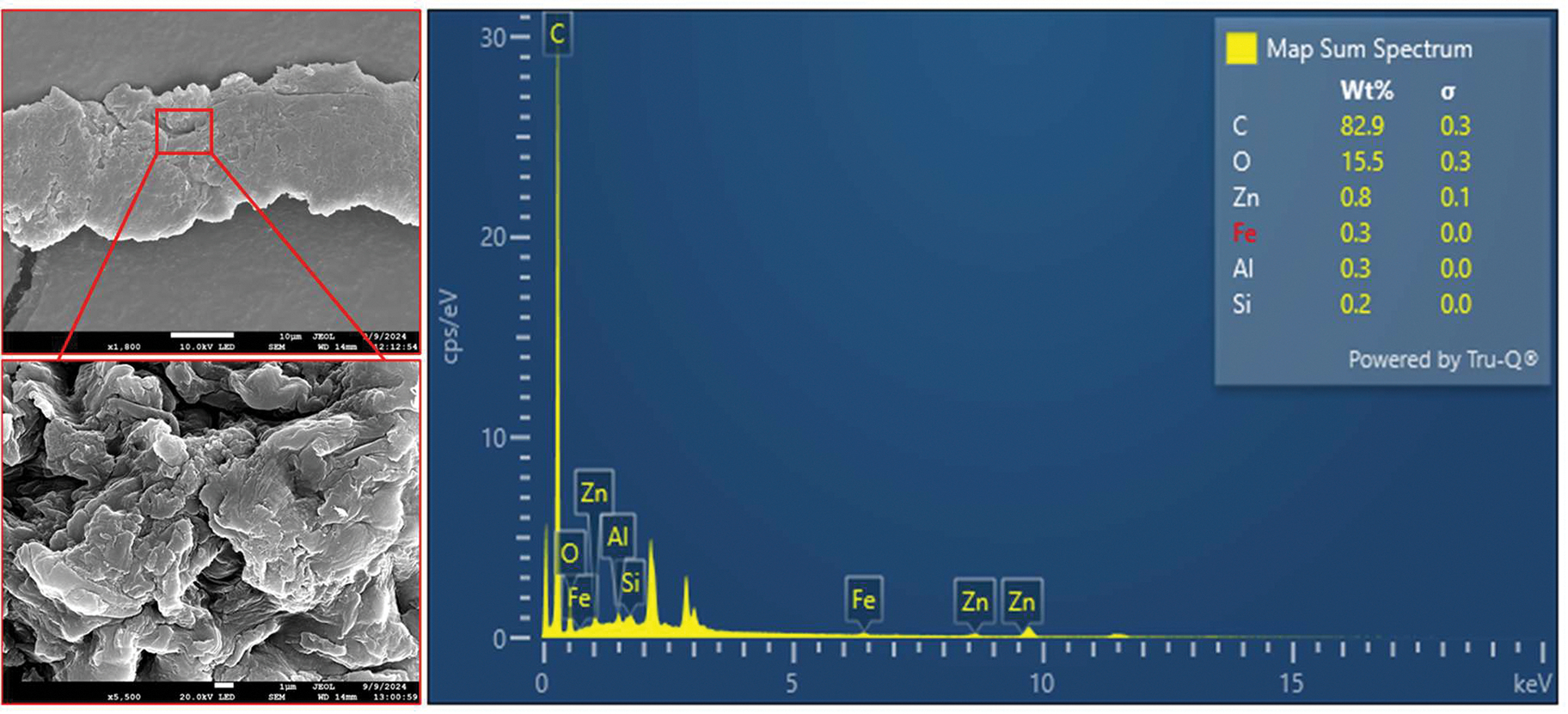
SEM-EDX analysis of a TWP from stormwater runoff. SEM images at 1800× and 5500× magnification (left) show features of a TWP (elongated shape, jagged edges, and rough surfaces). EDX analysis reveals the particle elemental composition, including Zn, Fe, Al, and Si, which are common markers for tire tread.

SEM-EDX analysis of putative TWPs from the stormwater samples shows both morpho-textual features and elemental fingerprints associated with TWPs (Figure 3). For example, in particles resembling TWPs, there were significant concentrations of Zn and Fe, which are commonly used as markers for tires [17,75]. To further validate the identity of select putative TWPs, μ-ATR-FTIR analysis (Section 2.4) confirmed the presence of isobutylene isoprene rubber, a common synthetic elastomer used in the manufacturing of tires, in all ten of the analyzed TWPs.

### 3.2. Reduction in TWPs in Stormwater Runoff

**Figure 4.**
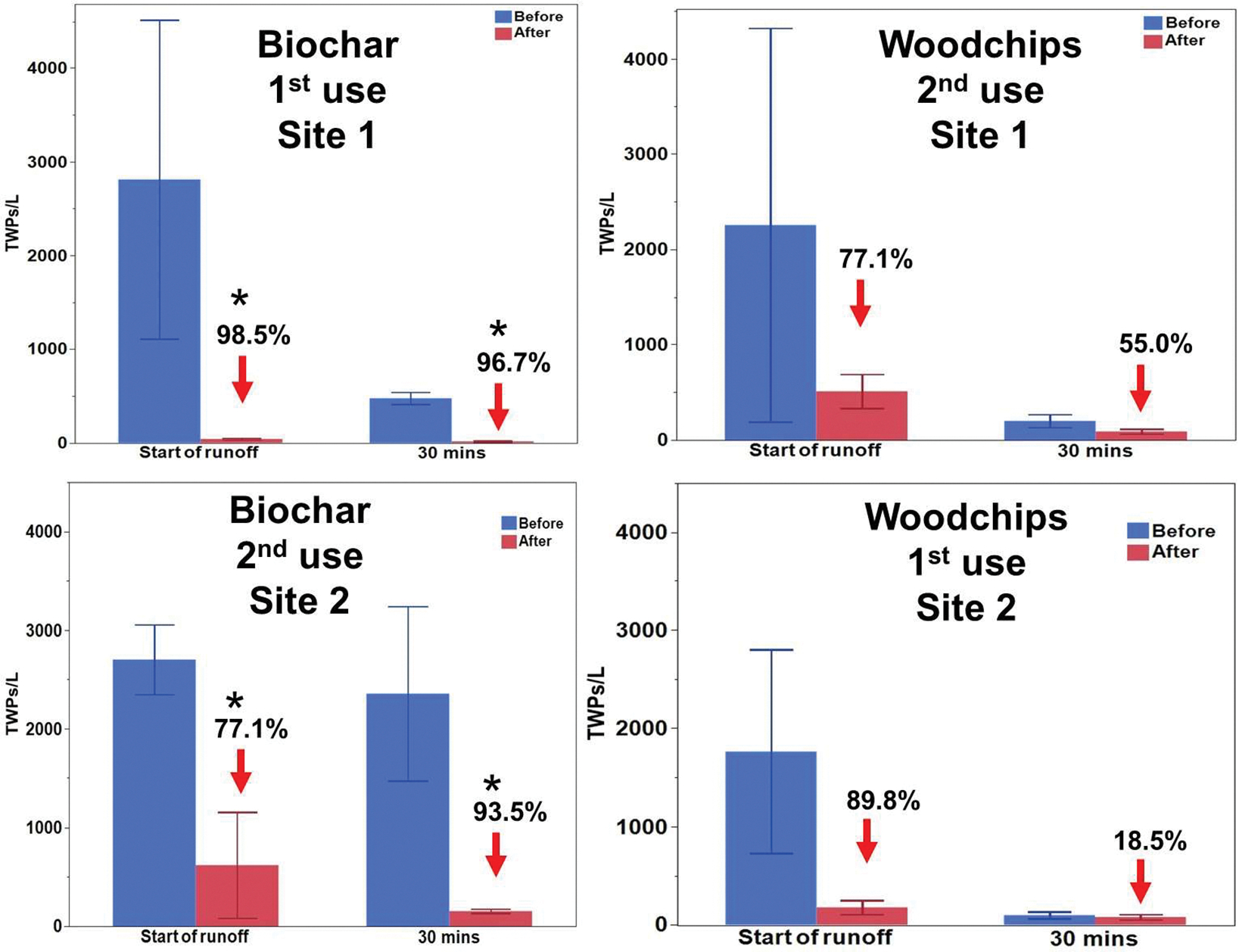
Plot showing average concentration (TWPs/L) of TWPs in stormwater runoff pre- and post-filter socks for biochar (**left**) and woodchips (**right**) for both sampling sites. The star (*) represents a significant difference at *p* < 0.05 level.

Whereas both the biochar and woodchip filter socks substantially reduced concentrations of TWPs from runoff, the reduction was only significant (*p* < 0.05) for the biochar (Figure 4). The details and discussion are provided in separate sections for biochar and woodchips below.

#### 3.2.1. Removal of TWPs with the Biochar Filter Sock

Paired *t*-tests confirmed statistically significant differences (*p* < 0.05) between pre- and post-biochar filtered samples, both at the onset of runoff and 30 min later (Figure 4, Table 3). At the start of the first storm event at site 1, the average TWP concentration (TWPs/L) in the runoff upstream of the biochar sock was 2811 ± 1700. This decreased to 40 ± 9.6 after filtering through the biochar filter sock, a reduction of 98.5%. Samples collected 30 min into the storm event followed a comparable trend, with concentrations decreasing from an average of 476.3 ± 63 before filtration to 15.3 ± 6 after the biochar filter socks, marking a 96.7% reduction. Overall, the biochar had a total average reduction of 97.6%. Additionally, a Kruskal–Wallis one-way ANOVA revealed significant differences among the datasets for biochar and woodchip biofilters at sampling site 2 across all sampling time points (H = 20.933, *p* = 0.004). However, pairwise multiple comparisons using Tukey’s post hoc test were unable to determine which groups were different, as it showed no significant differences between datasets for “before biochar” and “after biochar” at each time point (*p* > 0.05). This suggests that the variability within each dataset was high relative to the differences between the “before” and “after” groups. This could be due to natural fluctuations in TWP concentrations in the runoff, variations in biochar performance over time, or the presence of other environmental factors affecting particle transport and retention. Because of the high variability between grab samples, triplicate sampling may not provide sufficient statistical power to distinguish between individual groups effectively. Thus, we suggest caution interpreting the ANOVA results.

For the second storm event, the biochar filter was swapped to sampling site 2 to assess its performance across different sites. After the swap, similar trends in TWP reduction were observed. At the start of the runoff, the average TWP concentration (TWPs/L) prior to filtration was 2702 ± 353, which significantly decreased to 619 ± 536 after passing through the biochar filter, representing a reduction of 77.1%. Likewise, samples collected 30 min into the storm event showed a substantial decline in TWP concentrations, dropping from an average of 2356 ± 884 before filtration to 153 ± 22 after filtration, corresponding to a reduction of 93.5% and a total average reduction of 85.3% (Figure 4, Table 3). Paired *t*-tests conducted on the dataset indicated statistically significant differences in TWP concentrations before and after filtration, both at the start of the runoff (*p* = 0.04) and 30 min into the storm (*p* = 0.02). Similar to the results from sampling site 1, a Kruskal–Wallis one-way ANOVA revealed significant differences among all data groups for biochar and woodchip biofilters across all sampling time points at site 2 (H = 18.413, *p* = 0.010). However, pairwise comparisons using Tukey’s post hoc test showed no statistically significant differences between the independent datasets for “before” and “after” at each time point (*p* > 0.05).

**Figure 5.**
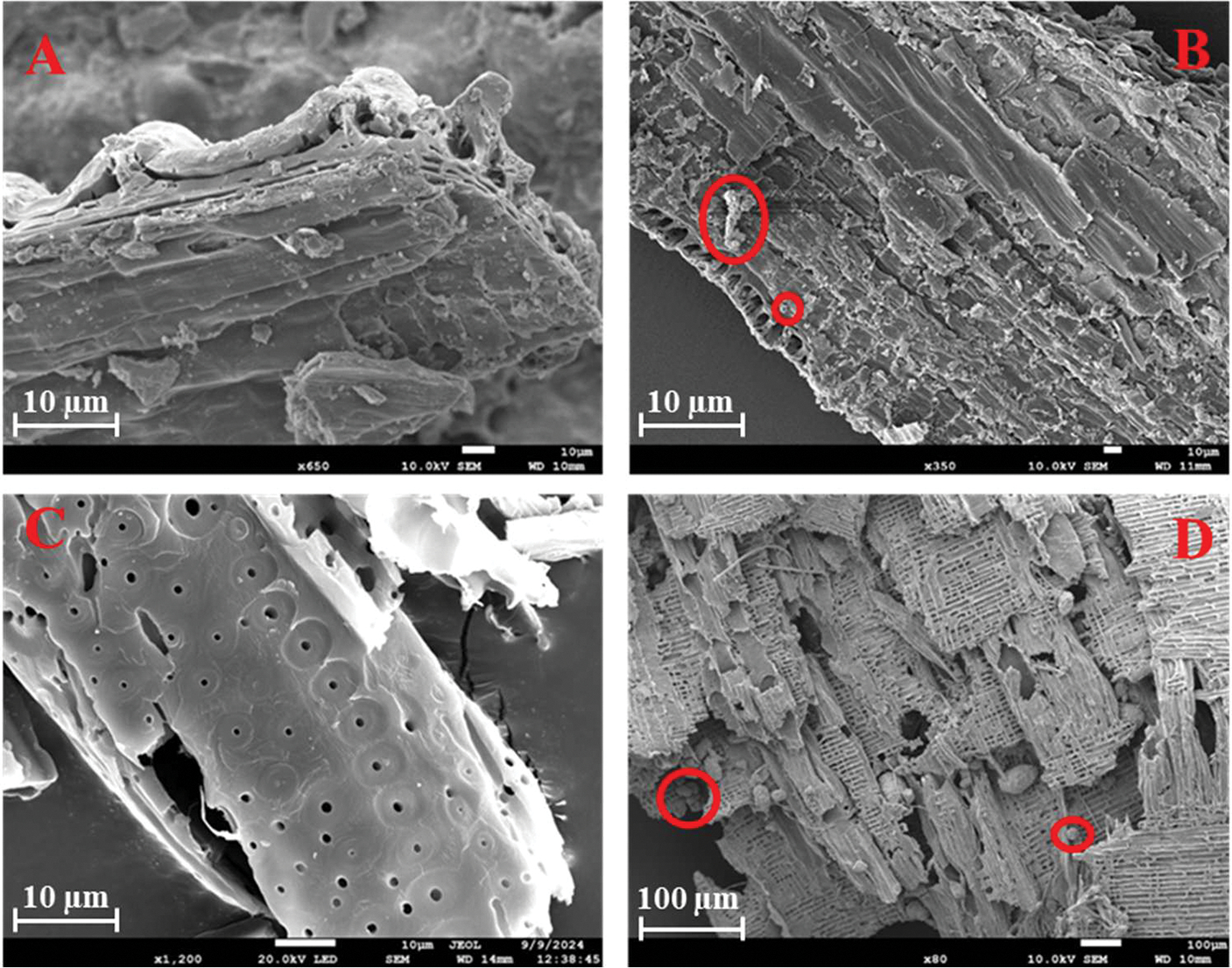
SEM images of rice husk biochar before interacting with stormwater runoff (**A**) and after a storm event (**B**) and similar images for pinewood woodchips before (**C**) and after (**D**). Note particles trapped or on the surface of the materials circled in (**B**,**D**).

Biochar’s physicochemical properties make it suitable for removing a variety of chemical pollutants from runoff through adsorption, electrostatic interactions, hydrogen bonding, hydrophobic interactions, and π–π stacking interactions [76–78]. Unlike chemical constituents, jagged TWPs are more likely to be physically entangled in the porous structure, though some chemical interactions may also occur. Therefore, pore filling and physical entrapment are likely the predominant mechanisms for TWP retention. Others have also reported that MPs can be trapped in biochar pores [43,79]. Here, the SEM images of rice husk biochar recovered from the field after the storm events revealed particles resembling TWPs and MPs embedded in the porous biochar matrix (Figure 5B).

#### 3.2.2. Removal of TWPs with the Woodchips Filter Sock

For the woodchips, similar trends were observed (Figure 4). At sampling site 1, at the start of the storm, the average TWP concentration (TWPs/L) before filtration was 2255 ± 2064 compared to 511 ± 176 after passing through the woodchip filter socks, a reduction of 77.3%. However, after 30 min, the TWP concentrations decreased from only 200 ± 67 before filtration to 90 ± 24 after filtration, a reduction of 55% at this time point. Overall, the woodchip biofilter had an average reduction of 66.2% during the first flush. Paired *t*-tests revealed no statistically significant differences (*p* > 0.05) between pre- and post-filtration TWP concentrations, both at the onset of runoff and after 30 min.

At sampling site 2, at the start of the storm, the average TWP concentration before filtration was 1765 ± 1037 compared to 179 ± 71 after passing through the woodchip filter sock, a reduction of 88.9%. After 30 min, the TWP concentration decreased from 99 ± 36 before filtration to 81 ± 26 after filtration, a reduction of only 18.5% (Figure 4, Table 3). Overall, the woodchip biofilter had a total average reduction of 54.2%. Paired *t*-tests similarly showed no statistically significant differences (*p* > 0.05) between “before” and “after” datasets at both sampling sites.

Although each biofilter material demonstrated potential in reducing TWPs in stormwater runoff, the woodchip biofilter was generally less effective than biochar in retaining captured TWPs. Moreover, its effectiveness dropped off to a greater extent than the biochar during the 30 min sampling point, suggesting both fewer trapping sites initially and more tendency for the increased potential for saturation of retention sites.

Particle size distribution (PSD) of TWPs revealed that most particles ranged between 30 and 290 μm. This size range may influence the filtration performance of woodchips, as larger particles are more likely to be retained at the start of the storm, whereas smaller particles may bypass the filter as its retention capacity diminishes. Environmental variability is common in stormwater runoff studies due to inconsistent rain intensities, varying antecedent dry days, and differences in runoff volume, leading to non-normal data distributions.

#### 3.2.3. Biochar vs. Woodchips

**Figure 6.**
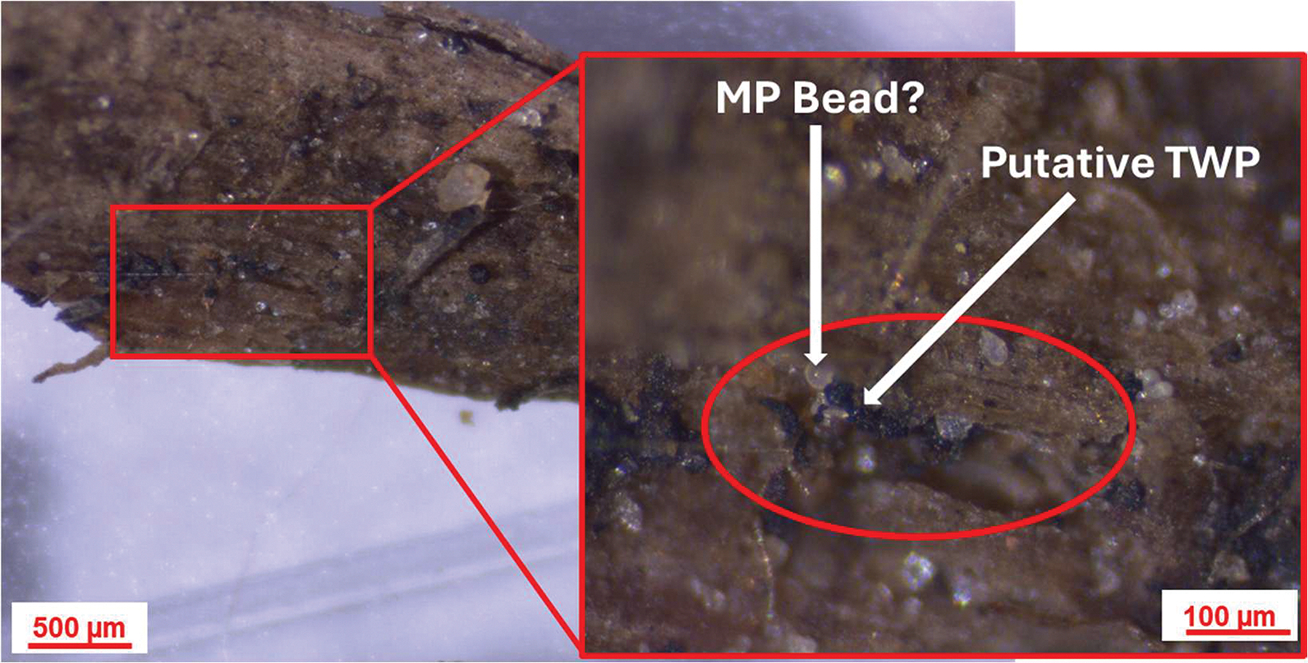
Optical images of woodchips at 40× (**left**) and 100× (**right**) magnification after deployment showing putative TWPs and possibly a microplastic bead trapped in the matrix.

Rice husk biochar was tightly packed in the filter socks due to its smaller and relatively uniform sizes, minimizing gaps and maximizing contact with stormwater runoff. In contrast, woodchips were more loosely packed, leaving some gaps between the individual pieces. These gaps likely allowed for pollutants, including TWPs, to flow through more easily, reducing the overall filtration efficiency. Additionally, structural changes in the woodchips were observed during the storm events. Pores within the woodchips expanded upon interacting with stormwater runoff during the “first flush effect”, altering their characteristics and trapping efficiency (Figure 5). Woodchips share similar characteristics with bark, as both exhibit high porosity and likely contain organic compounds, such as cellulose, lignin, and tannin [56,80]. Lignin features both aromatic and hydrophobic surface groups, which may aid in the removal of pollutants with hydrophilic and hydrophobic properties [81]. Others have shown that wood residues, such as sawdust, coated with natural phenolic compounds effectively captured MPs [58]. This removal efficiency was attributed to surface adsorption facilitated by complex multi-molecular interactions in vertical filtration systems. While this study employed a horizontal flow system in the field, the SEM and stereomicroscopic images of the recovered woodchips revealed significant pore expansion and particle accumulation on their surfaces (Figures 5 and 6). This suggests pore filling as well as surface adsorption facilitated by the aromatic and hydrophobic groups associated with the lignin content of the woodchips are potential mechanisms of interaction. However, our results show that the woodchip’s capacity was reduced with time during the storm event, suggesting that, as the woodchip pores and surface became saturated with TWPs and other materials, the filtration capacity diminished. By the 30 min mark, most of the available trapping sites on the woodchip’s surface were already occupied, leaving fewer pores for additional TWPs to be retained. This saturation effect possibly explains the reduced filtration efficiency of the woodchip biofilters over time.

In contrast, biochar demonstrated superior performance due to its high surface area and porous structure (Table 1). Previous studies have reported that biochar generated at elevated temperatures typically exhibits a greater surface area and carbon content, predominantly attributed to the elimination of volatile organic compounds during the pyrolysis, increasing in micropore volume [51]. Similarly, other studies have shown that pyrolysis temperature can affect sorption mechanisms. At low pyrolysis temperatures (<400 °C), partitioning is the main mechanism, whereas at high pyrolysis temperatures (>500 °C), adsorption is the primary mechanism [52]. The carbonized portion of the biochar acts as an adsorbent, attracting contaminants through electrostatic forces and non-polar interactions, while the non-carbonized portion acts as a partitioning mediator [52]. Based on our observations in this study, rice husk biochar, produced at a higher pyrolysis temperature (600 °C), exhibits an increased porosity (Table 1). These surface pores play a crucial role in trapping TWPs and MPs from runoff as they pass through the biochar, employing a “trap-and-stuck” mechanism (Figure 5B) described in Wang et al. [53]. MPs and TWPs have distinct morphologies. MPs are typically categorized as beads, fragments, films, or fibers [43], whereas TWPs have an elongated shape and a rougher and more irregular surface with numerous cavities [82]. These structural differences influence their fate and transport behavior in runoff. The elongated shape of TWPs increases their likelihood of becoming trapped within the pores of the biofilters, enhancing their retention compared to other adsorption mechanisms. This pore-filling mechanism in addition to physical entrapment likely allowed for rice husk biochar pyrolyzed slowly at 600 °C to trap TWPs effectively and maintain its filtration efficiency even during prolonged exposure to stormwater runoff. Unlike woodchips, the densely packed biochar filter socks provided consistent contaminant retention, preventing TWPs from bypassing the filtration medium.

The reduced effectiveness of woodchip biofilters over extended periods could also be attributed to differences in their physicochemical properties compared to biochar. Rice husk biochar’s superior performance in this study may be due to its higher micropore volume and adsorption capacity, enhanced by its carbon-rich composition and functional groups that facilitate binding with TWPs. Woodchips, while effective initially, may lack the same properties as the biochar, leading to faster declines in filtration efficiency. Thus, understanding the mechanics of filtration within these materials is essential for improving the design of stormwater management systems. Biochar’s consistently superior results suggest that it is a more promising solution for reducing TWPs in stormwater runoff. These findings emphasize the importance of selecting filtration materials based on stormwater flow conditions and contaminant retention requirements. While woodchips may be suitable for short-term or low-intensity runoff events, biochar appears to be a more robust and sustainable option for prolonged or high-intensity runoff, offering greater reductions in TWP concentrations and long-term contaminant trapping efficiency.

### 3.3. Particle Size Distribution (PSD) of TWPs in Urban Stormwater Runoff

**Figure 7.**
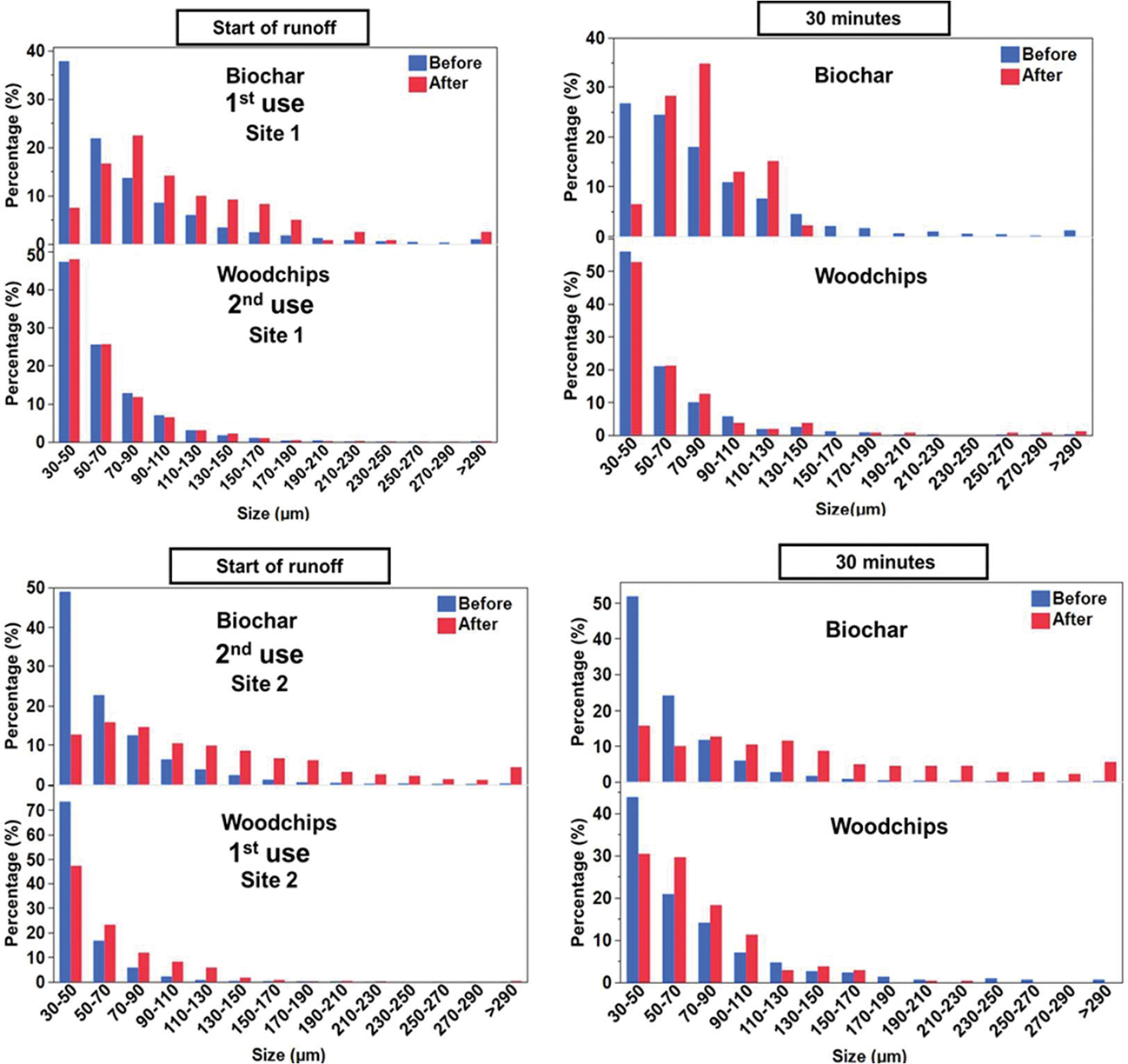
Plot showing particle size distribution of TWPs in stormwater runoff before and after treatment with the biochar and woodchip filter socks at site 1 (**top**) and site 2 (**bottom**). Data for the start of the runoff are presented at the left, and data for the 30 min time point are presented at the right. Note: The figure is based on percentages of absolute quantities to best show the change in particle size distribution between groups. The number of particles is plotted in Figure 4.

Smaller-sized TWPs are more likely to be transported over greater distances compared to larger particles, which more readily settle in drainage channels and gullies [83]. As such, it is important to characterize the size of TWPs, both in the initial stormwater runoff and post-filtration by both the biochar and woodchips. In this study, the majority of the TWPs were between 30 and 70 μm, with an average particle size of ~90 μm (Figure 7). It is important to note that particles <30 μm were excluded from this study due to sample preparation procedures (see Section 2.3). For biochar, the smaller particle size fractions (30–70 μm) showed a decreased percentage of particles after filtration. However, the percentage of particles increased after filtration for the larger particle size fractions (>90 μm) (Figure 7). For woodchips, the first use had a similar pattern to the biochar, albeit to a lesser extent, but that changed in the second use, where the distribution pattern was essentially the same for both before and after samples. This suggests that biochar was particularly effective at removing smaller particles, perhaps due to its larger surface area and pores, as can be seen in Figure 5, and that woodchips can also trap smaller particles but become less effective with use, possibly reflecting saturated trapping sites.

We have previously characterized airborne TWPs near the same site and found a similar if not slightly lower size distribution [23]. Kreider et al. [13] reported a size range of 4–265 μm, with a mode of 25 μm (after sieving samples at 150 μm), and Kovochich et al. [84] identified TWPs in road dust with an average particle size of 158 μm by number. The latter study also found that 62% of particles were elongated (aspect ratio > 1.5) within a size range of 6–649 μm (n = 113). The PSD of TWPs collected near roadways can vary significantly due to vehicle speed, driving conditions, tire composition, road surface characteristics, and climatic conditions [85]. Additionally, analytical methods employed for the collection and processing of TWPs can contribute to variations in size distribution [18,86,87]. Overall, PSD analysis in this study demonstrates that biofilters used, particularly biochar, are potentially effective in capturing and removing TWPs of various size fractions, especially the smaller size fraction, from urban stormwater runoff near roadways.

### 3.4. Study Limitations

This study was conducted on a limited scale, with biofilters deployed along a specific roadway and assessed over two storm events in June and July 2024. The short duration of the storms and small-scale setup may not fully represent year-round or large-scale applications, limiting the generalizability of the findings to other environments and extended time frames. Factors such as varying rainfall intensity, storm duration, and the number of dry days preceding storm events impacted the concentration and behavior of TWPs in runoff. As a result, these environmental variables could introduce inconsistencies in TWP retention performance, and additional studies are needed to assess the effectiveness of biochar and woodchips under diverse weather patterns.

Additionally, biochar and woodchip biofilters may experience clogging over time, especially in high TWP concentration areas, which could reduce their filtration efficiency. Here, we did not evaluate long-term performance, maintenance needs, or end-of-life cycle considerations, all of which are critical for sustained application in urban stormwater systems. While our study demonstrated effectiveness in capturing TWPs, it did not evaluate the biofilters’ performance on other contaminants commonly found in roadway runoff, such as MPs, trace metals, and per- and polyfluoroalkyl substances (PFAS). Interestingly, Ihenetu et al. [88] recently reported that biochar effectively reduced TWP leachates (6PPD and 6PPD-Q) and antimony (Sb) bioavailability in soils. As such, this biofiltration media’s effectiveness against a broader range of contaminants should be investigated to assess their versatility in stormwater management.

Finally, bulk sampling using glass jars, which capture all particles regardless of their size, was used to collect the runoff samples in this study. While this approach is cost-effective, it provides only a snapshot of MP and TWP abundance at a specific time and location, introducing higher variability [89]. Also, the effectiveness of biochar and woodchips may vary with local soil composition, roadway traffic, and existing urban infrastructure, which were specific to the study site. Replicating this study across different regions with different sampling approaches and varied environmental conditions and traffic patterns would provide a more robust evaluation of biochar and woodchips as biofilters in diverse urban landscapes.

## 4. Conclusions

TWPs, which frequently end up in receiving waterbodies, have been shown to pose serious risks to aquatic life due, in part, to the leaching of toxic compounds like 6PPD-Q. Here, we evaluated the potential of biochar and woodchips in stormwater filtration socks to mitigate the transfer of TWPs from urban runoff into aquatic environments. The results of our field study are promising, showing a significant reduction in TWP concentrations after stormwater runoff passed through the filtration media. Biochar proved to be more effective in capturing TWPs across the two sampling sites, with a removal rate >85% compared to <66% for woodchips. Biochar’s unique physicochemical properties, including its high surface area and adsorption, abundant pores, and numerous surface functional groups, give it adsorption capacity, making it suitable for trapping contaminants in stormwater, including TWPs. Thus, it has the potential to mitigate the spread of pollution to downstream environments. Moreover, biochar and woodchips are environmentally benign low-cost material that can be sourced from agroecological waste. Future studies on this biofiltration approach for TWPs should focus on repeatability, scaling, and improved design and application in areas of heavy traffic as well its effectiveness for other contaminants in urban stormwater runoff, such as MPs and PFAS.

## Figures and Tables

**Table 1.** Physical and chemical properties of the rice husk biochar.

Pyrolysis temperature	600 °C
Carbon (weight %)	38.4%
Ash (weight %)	53.5%
S_BET_ (m^2^ g^−1^)	300
External surface area (m^2^ g^−1^)	12.71
Micropore volume W_0_ (cm^3^ g^−1^)	18.07
Average pore diameter (Å)	0.12

Source: Singh et al. [60].

**Table 2.** Sample information and estimated peak runoff flow during sampling at the start of the storm event.

Sampling Date	Prior Dry Days	Biofilter		Flow Rate (L/min)	

		Site 1	Site 2	Site 1	Site 2

26 June 2024	22	Biochar	Woodchip	24 ± 1.8	14 ± 0.3
25 July 2024	28	Woodchip	Biochar	32 ± 0.2	15 ± 0.5

**Table 3.** Summary of results showing concentrations of TWP in runoff before and after biochar and woodchip biofilters along with percent decreases at two sampling sites for two separate storm events.

Biofilter	Parameters	Sampling Site 1				Sampling Site 2			
		Event 1				Event 2			
		Start of runoff		30 min		Start of runoff		30 min	
		Before	After	Before	After	Before	After	Before	After
Biochar	Total TWPs	8433	120	1429	46	8104	1454	7068	452
	Mean ± SD	2811 ± 1700	40 ± 9.6	476 ± 63	15 ± 6	2702 ± 353	619 ± 536	2356 ± 884	153 ± 22
	*p*-value	0.01		0.01		0.04		0.02	
	% reduction	98.5%		96.7%		77.3%		93.5%	
	Parameters	Event 2				Event 1			
		Start of runoff		30 min		Start of runoff		30 min	
		Before	After	Before	After	Before	After	Before	After
Woodchip	Total TWPs	6765	1532	600	269	5296	537	297	240
	Mean ± SD	2255 ± 2064	511 ± 176	200 ± 67	90 ± 24	1765 ± 1037	179 ± 71	99 ± 36	81 ± 26
	*p*-value	0.13		0.08		0.10		0.10	
	% reduction	77.1%		55.0%		89.8%		18.5%	

## Data Availability

Dataset available on request from the authors.
